# Measuring higher order ambiguity preferences

**DOI:** 10.1007/s10683-017-9542-3

**Published:** 2017-08-31

**Authors:** Aurélien Baillon, Harris Schlesinger, Gijs van de Kuilen

**Affiliations:** 1 Erasmus University Rotterdam, Rotterdam, Netherlands; 2 University of Alabama, Tuscaloosa, USA; 3 Tilburg University, Tilburg, Netherlands

**Keywords:** Higher-order preferences, Prudence, Ambiguity attitude, Risk attitude, C91, D81

## Abstract

We report the results from an experiment designed to measure attitudes towards ambiguity beyond ambiguity aversion. In particular, we implement recently-proposed model-free preference conditions of ambiguity prudence and ambiguity temperance. Ambiguity prudence has been shown to play an important role in precautionary behavior and the mere presence of ambiguity averse agents in markets. We observe that the majority of individuals’ decisions are consistent with ambiguity aversion, ambiguity prudence and ambiguity temperance. This finding confirms the prediction of many popular (specifications of) ambiguity models and has important implications for models of prevention behavior.

**Electronic supplementary material:**

The online version of this article (doi:10.1007/s10683-017-9542-3) contains supplementary material, which is available to authorized users.

## Introduction

Risk aversion governs many parts of economic behavior. However, it has to be complemented by the higher-order risk preferences *prudence* and *temperance* if one wants to explain important economic phenomena such as precautionary saving or prevention efforts. Consider a decision maker (DM) whose income is the same now and at a future point in time, and who just learned that she will face a zero-mean risk at the later point in time. According to expected utility (EU) theory, the DM should reduce current consumption and increase savings (i.e., save precautionary) if his future expected marginal utility is higher than his present marginal utility, i.e. if he has a convex marginal utility (*u*′′′ > 0), a property typically called (risk) prudence (Leland 1968; Kimball 1990). This result illustrates the importance of risk attitudes in economic decision making beyond risk aversion.

For long, the literature on higher order risk preferences remained purely theoretical (Leland 1968; Sandmo 1970; Kimball 1990, 1993). Eeckhoudt and Schlesinger (2006) were the first to propose behavioral, testable conditions of higher order risk preferences that made experimental investigation of these attitudes possible. For instance, one can test whether the DM is risk prudent if he prefers adding a zero-mean risk to a high income state rather than to a low income state. The DM is risk temperate (fourth order, *u* ^(4)^ < 0 under EU) if he prefers to disaggregate independent zero-mean risks across equiprobable states of nature. The conditions proposed by Eeckhoudt and Schlesinger (2006) led to many measurements in the laboratory and in the field, generally finding strong evidence for risk prudence and, to a lesser extent, for risk temperance (Tarazona-Gomez 2004; Deck and Schlesinger 2010, 2014; Ebert and Wiesen 2011, 2014; Noussair et al. 2014; Maier and Rüger 2012).

The literature mentioned above only concerns objectively known probabilities. However, such probabilities are unavailable in many important economic situations (Knight 1921). People have been found to dislike the absence of objective probabilities, exhibiting ambiguity aversion. Since Ellsberg (1961) proposed his famous paradox, empirical studies of ambiguity preferences have virtually all been restricted to studying the causes and prevalence of ambiguity aversion (e.g., see Trautmann and van de Kuilen 2015).

This paper explores attitudes toward ambiguity beyond aversion. *Ambiguity prudence* is a preference for combining ambiguity with states of the world yielding a high chance of a good outcome, rather than with states yielding a low chance of a good outcome. An *ambiguity temperate* DM dislikes facing two sources of ambiguity at the same time, and prefers to disaggregate them (for details, see Sect. 2).

Experimental evidence about higher order ambiguity attitudes is necessary to complement the recent theoretical literature that highlighted the importance of these concepts. For example, ambiguity prudence has been found to play a key role in models of survival of ambiguity averse agents on a market (Guerdjikova and Sciubba 2015) and of prevention behavior (Baillon 2017). Ambiguity prudence is necessary and sufficient for future ambiguity to trigger an increase in prevention efforts, e.g., an ambiguity prudent DM would rather get a vaccine against a new (“ambiguous”) disease than against a well-known disease with the same average severity and incidence (Baillon 2017).^1^ Moreover, specifications of popular ambiguity models (Gilboa and Schmeidler 1989; Schmeidler 1989; Hansen and Sargent 2001; Ghirardato et al. 2004; Klibanoff et al. 2005) that are widely used in applications imply ambiguity prudence. A policy maker who makes *robust* decisions in the sense of Hansen and Sargent (2007) is ambiguity prudent and ambiguity temperate. Despite the theoretical importance of higher order ambiguity attitudes for economic decision making, there is no evidence regarding the prevalence of higher order ambiguity attitudes in the population.

This paper is the first to investigate higher-order ambiguity preferences empirically. First, we develop an experimental methodology to implement the preference conditions proposed by Baillon (2017). Second, we embed our tests in a more general experiment, also measuring ambiguity aversion and higher order risk preferences (risk aversion, risk prudence and risk temperance). This allows us to investigate the relationship between these attitudes, and to test whether both these preferences stem from combining good outcomes with bad outcomes, as recently conjectured by Crainich et al. (2013) and observed by Deck and Schlesinger (2014). Our results show clear support for ambiguity prudence, thereby confirming the predictions of several ambiguity models. Our findings further support, to a lesser extent, ambiguity temperance.

The remainder of this paper is organized as follows. In the next section, we present the theoretical foundation of our measurements of higher order ambiguity attitudes. Section 3 describes the design of our experiment. The results are presented in Sect. 4 and are discussed in Sect. 5. Section 6 concludes.

## Higher order preferences

### Order 2: aversion

A DM is facing a choice between two 50–50 lotteries A_r2_ ≡ (*x* – *k*_1_ – *k*_2_, *x*) and B_r2_ ≡ (*x* – *k*_1_, *x* – *k*_2_) for some *x*, *k*_1_, *k*_2_ > 0.^2^ In lottery B_r2_, the two losses (two “harms”) –*k*_1_ and –*k*_2_ are “disaggregated”, i.e., one of them occurs only if the other does not. By contrast, the harms are aggregated in lottery A_r2_. *Risk aversion* can be defined as preferring B_r2_ to A_r2_ for all *W*, *k*_1_, and *k*_2_. Hence, risk averters prefer disaggregating the two harms. Under EU, it is equivalent to *u*″ < 0. *Risk lovers* always prefer A_r2_ to B_r2_ and therefore satisfy *u*″ > 0 under EU. We used 50–50 lotteries in the definition to show the similarity with the definitions that will follow. Of course, A_r2_ can be any lottery and B_r2_ its expected value. We write (*p*: *x*, *y*) to refer to binary lotteries yielding *x* with probability *p* and *y* otherwise. If *p* is omitted, then it is 50%.

**Fig. 1:**
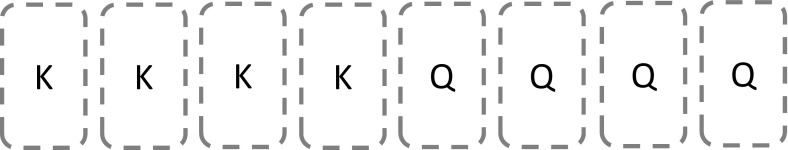
Deck of cards to test ambiguity aversion. *Note*: The dashed lines indicate that either all cards are red or all cards are black

Consider a DM facing the following variant of the Ellsberg paradox, based on the deck of 8 cards depicted in Fig. 1. Half of the cards are King, the other half are Queen. Yet, the color of the cards (red or black) is not known and neither is the process determining the color. This situation corresponds to ambiguity. The only information is that either all cards are red or all cards are black. The DM can pick a color, and wins €30 if he draws a card of the picked color (bet A) or he may win €30 if he draws a King from the deck (bet B). Hence, bet B gives 50% to win while bet A gives either 0 or 100%.

Let ({*p*, *q*}: *x*, *y*) describe a bet in which one state of nature (e.g., one possible composition of the deck) gives a probability *p* of winning *x* (and *y* < *x* otherwise) and the only other possible state of nature gives a probability *q* of winning the same prize *x* (and *y* otherwise). In the remainder, when using the notation {*p*, *q*}, we will assume *informational symmetry*: the DM does not have information favoring one of the two states over the other. Bet A can be represented by ({0%,100%}: €30, €0) and bet B by ({50%, 50%}: €30, €0) (whatever the state of nature, i.e., whatever the color composition of the deck, the probability of winning is 50%). Preferring bet B, with the known probability of winning *x*, to bet A is interpreted as ambiguity aversion. Consider the pair of bets A_a2_ and B_a2_ defined by A_a2_ ≡ ({*p* – *q*_1_ – *q*_2_, *p*}: *x*, *y*) and B_a2_ ≡ ({*p* – *q*_1_, *p* – *q*_2_}: *x*, *y*), with *x* > *y*, 0 ≤ *p* ≤ 1, *q*_1_ > 0, *q*_2_ > 0, and *p* – *q*_1_ – *q*_2_ ≥ 0. We call –*q*_1_ and –*q*_2_ *probability losses*, i.e. reductions of the probability of winning. *Ambiguity aversion* can be characterized as preferring B_a2_ to A_a2_ for all such pairs of bets. It can be seen as aversion toward mean preserving spreads in winning probabilities. The opposite preference is called *ambiguity loving*.

Many models have been proposed to accommodate ambiguity aversion. Among others, maxmin expected utility (Gilboa and Schmeidler 1989), Choquet expected utility with convex capacities (Schmeidler 1989), variational preferences (Maccheroni et al. 2006) all predict a preference for B_a2_ in the choice described above. Klibanoff et al. (2005) introduced a smooth model of ambiguity attitudes, which is close in spirit to EU. Consider a pair of bets A_a2_ and B_a2_. The smooth model involves three functions: a von Neumann and Morgenstern utility function *u* that we scale such that *u*(*y*) = 0 and *u*(*x*) = 1; a subjective probability function *μ* over the possible states of nature, and a smooth ambiguity attitude function *φ.* According to the smooth model, A_a2_ and B_a2_ are evaluated by *μ*(*p* – *q*_1_ – *q*_2_)*φ*(*p* – *q*_1_ – *q*_2_) + *μ*(*p*)*φ*(*p*) and *μ*(*p* – *q*_1_)*φ*(*p* – *q*_1_) + *μ*(*p* – *q*_2_)*φ*(*p* – *q*_2_) respectively. From informational symmetry, we obtain *μ*(*p*) = *μ*(*p* – *q*_1_ – *q*_2_) = *μ*(*p* – *q*_1_) = *μ*(*p* – *q*_2_) = 0.5 and therefore, that ambiguity aversion is equivalent to *φ*″ < 0.

### Order 3: prudence

Eeckhoudt and Schlesinger (2006) showed how to define higher order risk attitudes as a preference for disaggregating harms. Consider a choice between A_r3_ ≡ (*x*, *x* + $\tilde{\varepsilon }$_1_ – *k*_2_) and B_r3_ ≡ (*x* + $\tilde{\varepsilon }$_1_, *x* – *k*_2_), with $\tilde{\varepsilon }$_1_ a zero-mean risk. The two 50–50 lotteries involve a loss – *k*_2_ and another “harm”, the zero-mean risk $\tilde{\varepsilon }$_1_. *Risk prudence* can be defined as always preferring B_r3_ to A_r3_ for all *x*, $\tilde{\varepsilon }$_1_, and *k*_2_, hence preferring disaggregating the two harms. The DM would rather bear the loss in the situation in which he does not bear the risk. Under EU, it is equivalent to *u*′′′ > 0. A DM always preferring A_r3_ to B_r3_ is called *risk imprudent* and will satisfy *u*′′′ < 0.

**Fig. 2:**
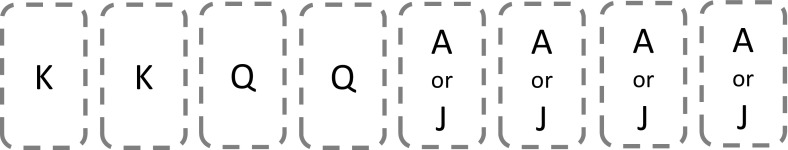
Deck of cards to test ambiguity prudence. *Note*: the dashed lines indicate that either all cards are red or all cards are black

Consider the deck depicted in Fig. 2, with 8 cards which are either all red or all black. Furthermore, we know that there are two kings and two queens in the deck, and that the four other cards are either all jacks or all aces. The DM wins €30 if he draws a king, a black queen or a red jack from the deck. This can be written as ({50%, 25% + {0%, 50%}}: €30, €0). In one state of nature (if the cards are black), he has a 50% chance of winning (there are kings and queens in the deck). In the other state of nature (red cards), he has a 25% chance of winning (there are two kings in the eight cards) *plus* an additional chance of winning depending on two independent states of nature, i.e., the absence or presence of jacks {0, 50%}. Now imagine that the DM must give up a possibility of winning: either he does not win if a red king is drawn *or* he does not win if a black king is drawn from the deck. If he gives up “red king” the bet becomes ({50%, 0% + {0%, 50%}}: €30, €0). If he gives up “black king” it becomes ({25%, 25% + {0%, 50%}}: €30, €0). In other words, the DM has to choose whether he should aggregate or disaggregate a 25% probability loss with the presence of ambiguity ({0, 50%}). An ambiguity prudent DM would rather disaggregate the two harms (probability loss and jack-related ambiguity) and therefore chooses to give up the black king.

Consider the general pair of bets A_a3_ and B_a3_ defined by A_a3_ ≡ ({*p*, *p* – *q*_2_ + {–*t*, *t*}}: *x*, *y*) and B_a3_ ≡ ({*p* – *q*_2_, *p* + {–*t*, *t*}}: *x*, *y*) with *x* > *y*, 0 ≤ *p* ≤ 1, *q*_2_ > 0, *t* > 0, *p* + *t* ≤ 1 and *p* – *q*_2_ – *t* ≥ 0. *Ambiguity prudence* is characterized by preferring B_a3_ to A_a3_ for all such pairs of bets. The opposite preference is called *ambiguity imprudence*. Baillon (2017) showed that ambiguity prudence is equivalent to *φ*′′′ > 0 in the smooth model and that some popular specifications of other ambiguity models predict ambiguity prudence: e.g., Hansen and Sargent’s (2001) multiplier preferences, Chateauneuf et al.’s (2007) Choquet expected utility with neoadditive capacities, maxmin expected utility (Gilboa and Schmeidler 1989) for some specific sets of priors.

### Order 4: temperance

To define the fourth order of risk attitude, let us consider two independent zero-mean risks $\tilde{\varepsilon }$_1_ and $\tilde{\varepsilon }$_2_ and define A_r4_ ≡ (*x*, *x* + $\tilde{\varepsilon }$_1_ + $\tilde{\varepsilon }$_2_) and B_r4_ ≡ (*x* + $\tilde{\varepsilon }$_1_, *x* + $\tilde{\varepsilon }$_2_). A DM is *risk temperate* if and only if he prefers B_r4_ to A_r4_ for all *x*, $\tilde{\varepsilon }$_1_, and $\tilde{\varepsilon }$_2_. Such a DM rather disaggregates the two (harmful) risks. The opposite preference is called *risk intemperance.* Risk (in)temperance is equivalent to *u* ^(4)^ < (>) 0 under EU (Eeckhoudt and Schlesinger 2006).

**Fig. 3:**
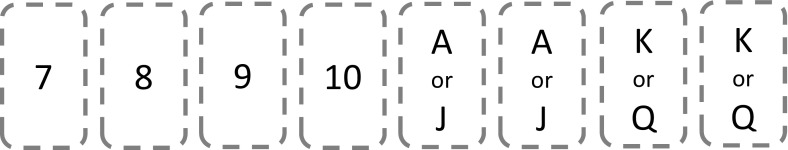
Deck of cards to test ambiguity temperance. *Note*: the dashed lines indicate that either all cards are red or all cards are black

Risk temperance is defined as a preference for disaggregating two independent zero-mean risks. Ambiguity temperance can similarly be defined as a preference for disaggregating two informationally-symmetric ambiguities. Consider the deck depicted in Fig. 3. Two cards may be aces and two may be kings. The DM wins €30 if he draws a red ace, a red king, a black 9 or a black 8 from the deck. This can be written as ({1/4, 1/4 + {–1/8, 1/8} + {–1/8, 1/8}}: €30, 0). In one state of nature (if the cards are black), the DM has a 25% chance of winning because there is one 9 and one 8 in the deck. In the other state of nature (red cards), the winning probability can be 0% (two jacks and two queens), 25% (two jacks and two kings or two aces and two queens), or 50% (two aces and two kings). Now imagine that the DM can choose to win €30 if he draws a red ace, a red 8, a black 9 or a black king from the deck instead. This can be written as ({1/4 + {–1/8, 1/8}, 1/4 + {–1/8, 1/8}}: €30, 0). Thus, the choice amounts to whether the DM would rather have the chance of winning depend on the presence of aces and kings if the cards are black, or have it depend on aces if the cards are black and kings if they are red (keeping the average chance of winning constant across colors). In other words, the DM has to choose if he prefers to aggregate or disaggregate the ambiguity regarding the presence of aces with the ambiguity regarding the presence of kings in the deck. An ambiguity temperate DM would rather disaggregate these informationally symmetric ambiguities, i.e., prefer ({1/4 + {–1/8, 1/8}, 1/4 + {–1/8, 1/8}}: €30, 0) over ({1/4, 1/4 + {–1/8, 1/8} + {–1/8, 1/8}}: €30, 0).

More generally, define a pair of bets A_a4_ and B_a4_ by A_a4_ ≡ ({*p*, *p* + {–*t*, *t*} + {–*s*, *s*}}: *x*, *y*) and B_a4_ ≡ ({*p* + {–*t*, *t*}, *p* + {–*s*, *s*}}: *x*, *y*) with *x* > *y*, 0 ≤ *p* ≤ 1, *t* > 0, *s* > 0, *p* + *t* + *s* ≤ 1 and *p* – *t* – *s* ≥ 0. *Ambiguity temperance* is defined as preferring B_a4_ to A_a__4_ for all such pairs of bets. The opposite preference is called *ambiguity intemperance*. Ambiguity temperance is equivalent to *φ* ^(4)^ < 0 in the smooth model.

In the definitions of ambiguity aversion, prudence, and temperance, we used binary spreads in probabilities (e.g. {–*t*, *t*}). Baillon (2017) showed that the definitions can also be adapted to series of such spreads. For instance, in the example for ambiguity prudence, we described a deck in which four cards (out of eight) were either jacks or aces and we denoted the ambiguity related to betting on jacks {0, 50%}. We can replace this example by a deck in which each of the four cards can be either an ace or a jack. In such a case, we will write [0, 50%], to indicate that intermediary probabilities are also possible.

### Mixed attitudes

We presented the risk apportionments of order 2, 3, and 4 and their equivalent for ambiguity, thereby illustrating the link between risk and ambiguity for each order. We now turn to the link between the various orders. The definitions of aversion, prudence, and temperance all relied on a preference for disaggregating harms. Under EU for risk, risk apportionment of order *n* for *n* = 2 to 4 is equivalent to *sgn u* ^(*n*)^ = (−1)^n+1^ as shown by Eeckhoudt and Schlesinger (2006). A function satisfying this property for all *n* was called *mixed risk averse* by Caballé and Pomansky (1996). Crainich et al. (2013) called *mixed risk loving* a utility function whose successive derivatives are all positive. They show that such a utility is equivalent to always preferring combining good with good (and bad with bad). At order 2, it implies risk seeking (preferring the aggregation of the losses in Fig. 1a). Hence, risks are “good.” At order 3, it surprisingly implies risk prudence because the DM prefers combining the “good” risk with the high outcome. At order 4, it implies risk intemperance (preferring the aggregation of the “good” risks).

Let us extend the analysis to the domain of ambiguity in the framework of the smooth model. In particular, let us call *mixed ambiguity averse* a smooth ambiguity function *φ* satisfying *sgn φ* ^(*n*)^ = (−1)^n+1^ and *mixed ambiguity loving* a smooth ambiguity function *φ* satisfying *sgn φ* ^(*n*)^ = 1. Mixed ambiguity aversion implies ambiguity aversion, prudence, and temperance and can be explained by a preference for combining good with bad. Mixed ambiguity loving implies ambiguity loving, prudence, and intemperance and can be explained by a preference for combining good with good.

If people either have a general tendency for combining good with bad or a tendency for combining good with good, then mixed risk averse (loving) DMs should also be mixed ambiguity averse (loving). Table 1 describes the two types of DMs that should then be observed.

**Table 1:** Combining good with bad or good with good

Prefer combining good with bad	Prefer combining good with good
Mixed risk averse Risk averse (*u*″ < 0) Risk prudent (*u*′′′> 0) Risk temperate (*u* ^(4)^ < 0)	Mixed risk loving Risk loving (*u*″> 0) Risk prudent (*u*′′′ > 0) Risk intemperate (*u* ^(4)^ > 0)
Mixed ambiguity averse Ambiguity averse (*φ*″ < 0) Ambiguity prudent (*φ*′′′> 0) Ambiguity temperate (*φ* ^(4)^ < 0)	Mixed ambiguity loving Ambiguity loving (*φ*″ > 0) Ambiguity prudent (*φ*′′′ > 0) Ambiguity intemperate (*φ* ^(4)^ > 0)

## Experimental design

One hundred ninety-nine students from Erasmus University Rotterdam participated in a computerized experiment, which was conducted at the ESE-econlab. Subjects were recruited among a pool of volunteers and were told the experiment could last up to 1 h 15 min. They were told they would receive a €5 participation fee and that they could additionally earn from €0 to €45. The subjects could expect earning €23.50 in total. Each session involved between 23 and 28 subjects. The mean age of subjects was 22 years, 62% were studying economics or finance (38% other social sciences), and 38% were female.

The experiment consisted of 2 parts and each part of 15 choices. One part involved the elicitation of attitudes towards risk, the other part involved the elicitation of attitudes towards ambiguity. Each part started with instructions, read out aloud by the experimenter. In four sessions, subjects first made the risky choices and then the ambiguity choices. In the other four sessions, the order of the parts was reversed. Within each part, the order of the choices varied across sessions, but this order was the same for all subjects in a given session.

The 30 choice tasks are described in Table 2. The tasks were selected to allow for comparability between the risk tasks and the ambiguity tasks. For instance, as Table 2 shows, options A in the tasks used to measure risk aversion (tasks 1–5) were used as options B in the tasks measuring ambiguity aversion (tasks 16–20).

**Table 2:** Choice tasks

Task	Dom	Order	Option A	Option B	EV
1	Risk	2	(1/2: €30, €0)	€15	€15
2	Risk	2	(1/2: €45, €15) = 1A + €15	1B + €15	€30
3	Risk	2	(1/2: €45, €0) = 1A × 1.5	1B × 1.5	€22.5
4	Risk	2	(1/3: €30, €0)	€10	€10
5	Risk	2	(2/3: €30, €0)	€20	€20
6	Risk	3	(1/2: €15, €7.5 + $\tilde{\varepsilon }$_1_) with $\tilde{\varepsilon }$_1_ = (1/2: €7.5, −€7.5)	(1/2: €15 + $\tilde{\varepsilon }$_1_, €7.5)	€11.25
7	Risk	3	6A + €15	6B + €15	€26.25
8	Risk	3	6A × 2	6B × 2	€22.5
9	Risk	3	(1/2: €10, €5 + $\tilde{\varepsilon }$_2_) with $\tilde{\varepsilon }$_2_ = (1/3: €10, −€5)	(1/2: €10 + $\tilde{\varepsilon }$_2_, €5)	€7.5
10	Risk	3	(1/2: €25, €10 + $\tilde{\varepsilon }$_3_) with $\tilde{\varepsilon }$_3_ = (2/3: €5, −€10)	(1/2: €25 + $\tilde{\varepsilon }$_3_, €10)	€17.5
11	Risk	4	(1/2: €15, €15 + $\tilde{\varepsilon }$_1_ + $\tilde{\varepsilon }$_1_)	(1/2: €15 + $\tilde{\varepsilon }$_1_, €15 + $\tilde{\varepsilon }$_1_)	€15
12	Risk	4	11A + €15	11B + €15	€30
13	Risk	4	11A × 1.5	11B × 1.5	€22.5
14	Risk	4	(1/2: €10, €10 + $\tilde{\varepsilon }$_2_ + $\tilde{\varepsilon }$_2_)	(1/2: €10 + $\tilde{\varepsilon }$_2_, €10 + $\tilde{\varepsilon }$_2_)	€10
15	Risk	4	(1/2: €20, €20 + $\tilde{\varepsilon }$_3_ + $\tilde{\varepsilon }$_3_)	(1/2: €20 + $\tilde{\varepsilon }$_3_, €20 + $\tilde{\varepsilon }$_3_)	€20
16	Amb	2	([0, 1]: €30, €0)	1A	€15
17	Amb	2	16A + €15	2A	€30
18	Amb	2	({0, 1}: €45, €0)	3A	€22.5
19	Amb	2	([0, 2/3]: €30, €0)	4A	€10
20	Amb	2	([1/3, 1]: €30, €0)	5A	€20
21	Amb	3	({1/2, 1/4 + [−1/4, 1/4]}: €30, €0)	({1/4, 1/2 + [−1/4, 1/4]}: €30, €0)	€11.25
22	Amb	3	21A + €15	21B + €15	€26.25
23	Amb	3	21A × 1.5	21B × 1.5	€16.88
24	Amb	3	({1/3, 1/6 + [−1/6, 1/6]}: €30, €0)	({1/6, 1/3 + [−1/6, 1/6]}: €30, €0)	€7.5
25	Amb	3	({2/3, 1/2 + [−1/6, 1/6]}: €30, €0)	({1/2, 2/3 + [−1/6, 1/6]}: €30, €0)	€17.5
26	Amb	4	({1/2, 1/2 + [−1/8, 1/8] + [−1/8, 1/8]}: €30, €0)	({1/2 + [−1/8, 1/8], 1/2 + [−1/8, 1/8]}: €30, €0)	€15
27	Amb	4	26A + €15	26B + €15	€30
28	Amb	4	26A × 1.5	26B × 1.5	€22.5
29	Amb	4	({1/3, 1/3 + [−1/6, 1/6] + [−1/6, 1/6]}: €30, €0)	({1/3 + [−1/6, 1/6], 1/3 + [−1/6, 1/6]}: €30, €0)	€10
30	Amb	4	({2/3, 2/3 + [−1/6, 1/6] + [−1/6, 1/6]}: €30, €0)	({2/3 + [−1/6, 1/6], 2/3 + [−1/6, 1/6]}: €30, €0)	€20

Table 1 gives, for each decision task, the two options subjects were asked to choose from. Tasks 1–15 are tasks related to risk attitude, and 16–30 to ambiguity attitude. The order (2 = aversion, 3 = prudence, or 4 = temperance) is reported in the third column. Column 4 describes option A and column 5 option B. The last column gives the expected value, which was the same for both options of a given task. For example, in Task 2, option B is described as 1B + €15, which means option B of Task 1 plus €15, so €30. Option A of Task 24 described as ({1/3, 1/6 + [−1/6, 1/6]}: €30, €0) yields €30 with a probability either of 1/3 or between 0 and 1/3

**Fig. 4:**
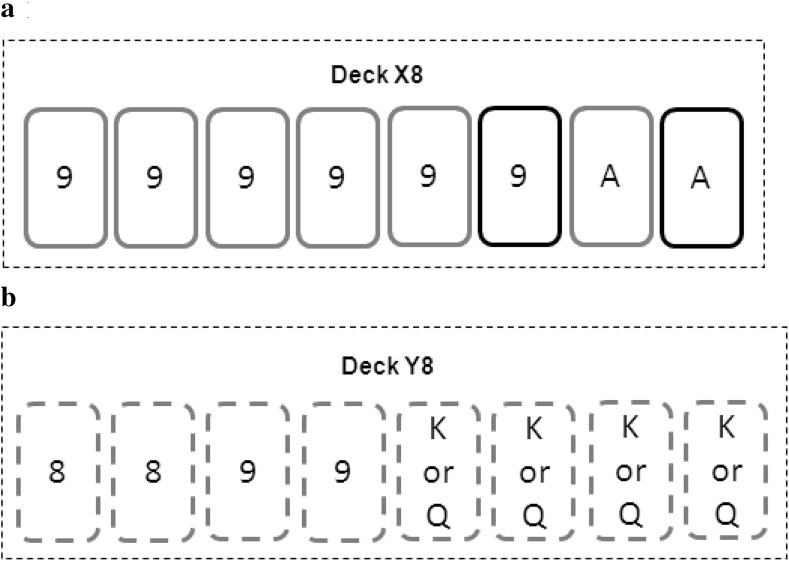
Decks for two prudence tasks. **a** Risk (task 8) and **b** Ambiguity (task 23)

Each experimental task corresponded to a deck of cards, which was prepared before the experiment and stored in a sealed envelope. The decks were described to the subject. For instance, Fig. 4 shows the deck of tasks 8 and 23 (from Table 2). For task 8 (Fig. 4a), six cards were red and two were black. Two cards were aces and six cards were 9 s. For task 23 (Fig. 4b), the deck contained eight cards, two of which were 8 s and two others were 9 s. The other four cards could be Kings or Queens, representing a first source of ambiguity. The dashed line depicting the cards indicated that it was unknown whether all cards were red or all cards were black, creating a second source of ambiguity.

**Fig. 5:**
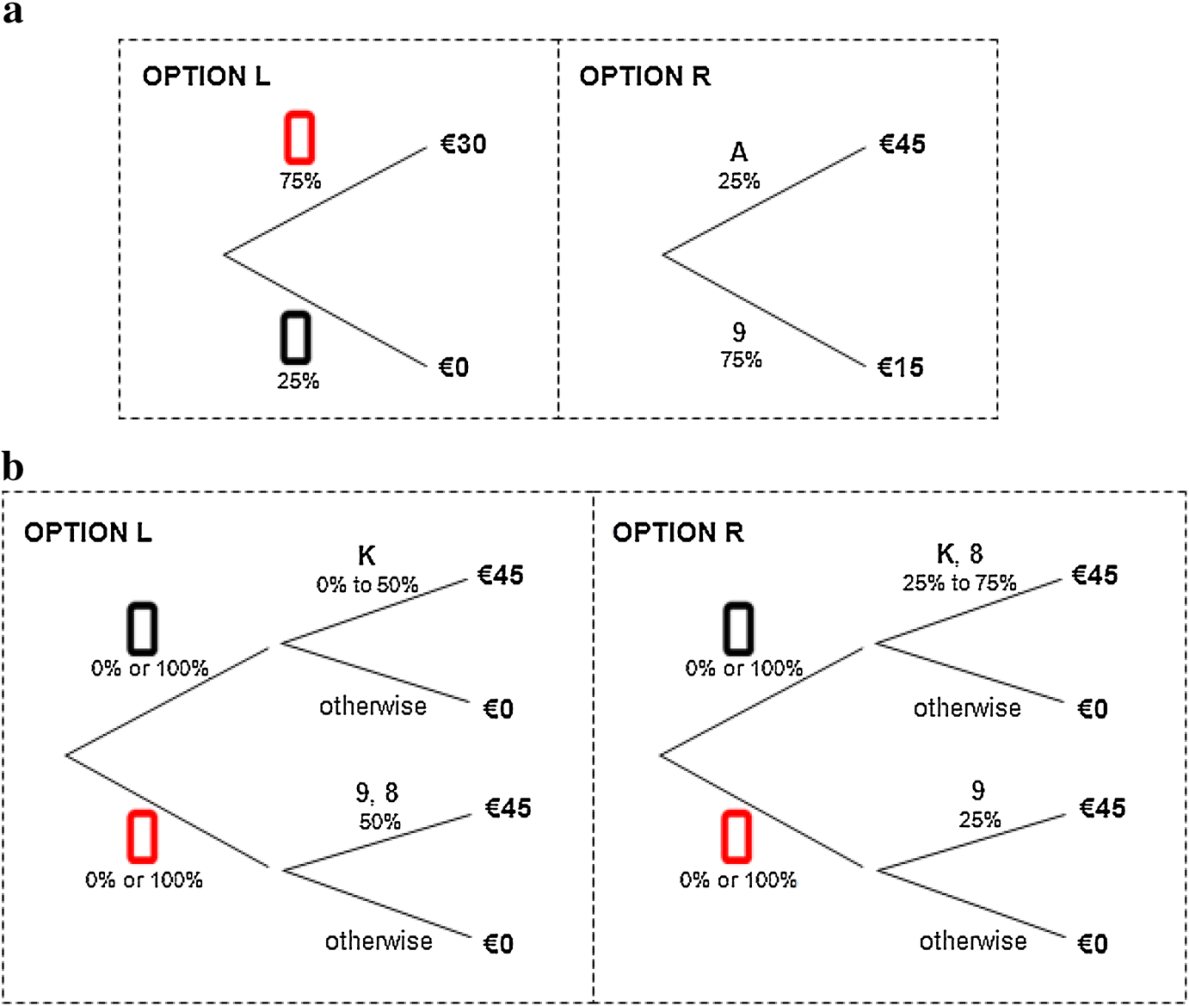
Choice options for two prudence tasks. **a** Risk prudence (task 8) and **b** Ambiguity prudence (task 23)

Together with Figs. 4a, and 5a was presented to subjects for task 8. In Fig. 5a, option A was presented as option L and option B as option R (in four of the eight sessions, L and R were reversed). With 50% chance, option A yielded €30 and with 50%, it yielded €15 and ±€15 (with equal probability). Lotteries were presented in a reduced form and therefore, the subjects were told that option L would give €30 with 75% chance and €0 otherwise.

Presenting reduced lotteries is a departure from most previous experimental investigations of higher order risk preferences. We did so because Halevy (2007) and Abdellaoui et al. (2015) found that ambiguity aversion is associated (strongly for the former study, weakly for the latter) with non-reduction of compound lotteries. Hence, we expected that presenting the lotteries in a non-reduced form could contaminate higher order risk preferences with violations of lottery reduction, and artificially inflate the association between higher order risk preferences and higher order ambiguity preferences. An important consequence of using reduced forms is that losses were not displayed as such but integrated into the final outcome (for instance, in Fig. 5a, the ±€15 risk never implies a net loss). Haering et al. (2017) found that DMs exhibit less prudence and temperance when lotteries are presented in their reduced form.

For task 23, the subjects saw on their computer screen both Figs. 4b and 5b (and some explanation). In both options, they would win €45 if a black King or a red 9 was drawn. In Option L (representing Option A of Table 2), they would also win if a red 8 was drawn and in Option R, if a black 8 was drawn. If the deck of Fig. 4b was composed of red cards, then Option L had a winning probability of 1/2, but if the deck is composed of black cards, the deck had a winning probability between 0 (if there was no king) and 1/2 (if there were four Kings). This implements ({1/2, 1/4 + [−1/4,1/4]}: €45, €0). Option R similarly implements ({1/4, 1/2 + [−1/4,1/4]}: €45, €0).

A potential problem in ambiguity tasks is suspicion: subjects may think that the experimenters have manipulated the likelihood that particular unknown events are realized to minimize the cost of the experiment. To avoid suspicion playing a role in the experiment, subjects were allowed to permute all ambiguous events. For example, in task 23, subjects could permute Kings and Queens and/or red and black. Since the decks were prepared before the experiment, the experimenters could not be suspected of strategic behavior. This possibility was also crucial to ensure that the subjects consider the various ambiguities to be informationally symmetric. They had no reason to believe that one color was more likely to be drawn than the other (see Sect. 3 for a further discussion).

Subjects were paid according to one of their choices in one randomly selected task. The implementation of random incentives can create issues in ambiguity experiments by offering the possibility for the subjects to hedge against ambiguity (e.g., Oechssler and Roomets 2014). In our experiment, each task concerned a different deck of cards and therefore, a different source of ambiguity. Hence, the random incentives did not provide a hedging possibility.

The incentives were implemented as follows. At the beginning of the session, one envelope was randomly drawn (by a subject) from a pile of 30 envelopes containing a decision task each. Subjects were told that the envelope (signed by the subject to identify it) would be opened at the end of the session and that the task it contains would be played for real money. This approach can enhance isolation (Johnson et al. 2014), making it clear that only one task is used for payment. Once the envelope was opened, the corresponding deck was mixed, and a subject drew a card from the deck. Payoffs were then determined and implemented.

## Results

### Order 2: aversion

**Fig. 6:**
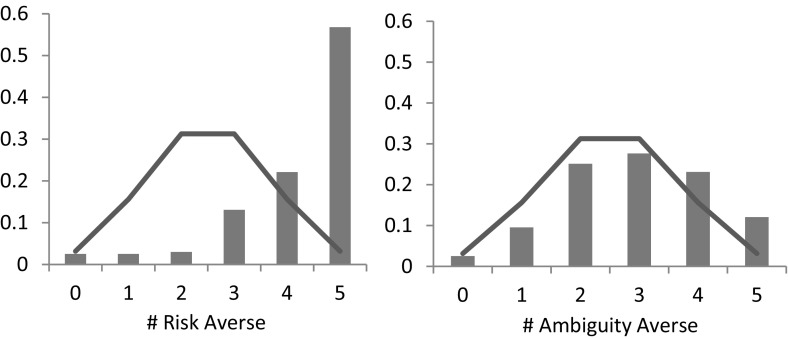
Distribution of risk- (*left*) and ambiguity- (*right*) averse choices. *Note*: The solid line indicates the frequency with which a given number of choices would be expected to occur if each subjects chose randomly

In line with Noussair et al. (2014) and Deck and Schlesinger (2014), we use the number of choices (out of 5) that subjects answered in a risk/ambiguity averse way as our (model-free) measurement of risk-and ambiguity preferences. Figure 6 presents the distribution of risk (left) and ambiguity (right) averse choices the 199 participants made in the experiment.

First, as the figure shows and in line with previous literature, the majority of choices in the experiment is consistent with risk aversion. On average, subjects chose the risk averse option 4.20 out of 5 times, which is significantly different from the average that would be observed if subjects chose randomly (Wilcoxon signed-rank test,^3^ *p* value < 0.01). More than half of the subjects (57%) chose the risk averse option in all 5 choices. In addition, the observed distribution of risk averse choices is significantly different what would be observed if subjects chose randomly (*χ* ^2^ test, *p*-value < 0.01).

As the right panel of the Fig. 6 suggests, we also observe ambiguity aversion. On average, subjects chose the ambiguity averse option 2.95 out of 5 times, which is significantly different from random choice (Wilcoxon signed-rank test, *p*-value < 0.01). In addition, the observed distribution of ambiguity averse choices is significantly different from the distribution that would be observed if subjects chose randomly (*χ* ^2^ test, *p*-value < 0.01).

**Fig. 7:**
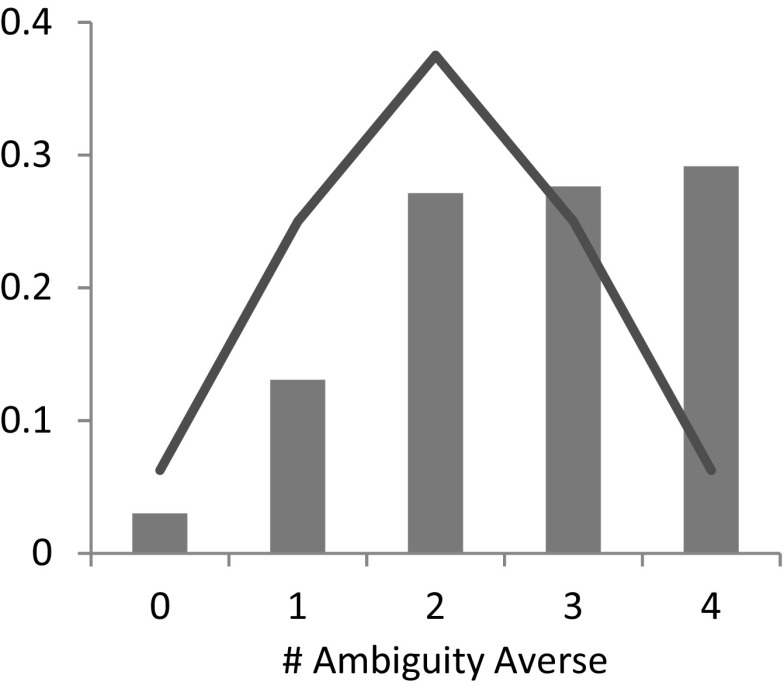
Distribution of ambiguity-averse choices for intermediate and high likelihoods. *Note*: The solid line indicates the frequency with which a given number of choices would be expected to occur if each subjects chose randomly

Note that ambiguity aversion appears to be less pronounced than risk aversion. The main reason for this finding is the fact that a vast majority (142 out of 199) of subjects preferred the ambiguity seeking option in Task 19, i.e., when the probability of winning was in [0, 2/3]. This finding corroborates the commonly found ambiguity seeking preference for lower likelihoods (see Trautmann and van de Kuilen 2015 for a summary of the experimental findings), and shows that assuming universal ambiguity aversion might be problematic from a descriptive point of view. Figure 7 displays the proportion of ambiguity averse choices excluding Task 19, i.e. for choices involving intermediate or high likelihoods. In this case, a majority of subjects (57%) chose the ambiguity averse lottery 3 or 4 times out of 4.

### Order 3: prudence

**Fig. 8:**
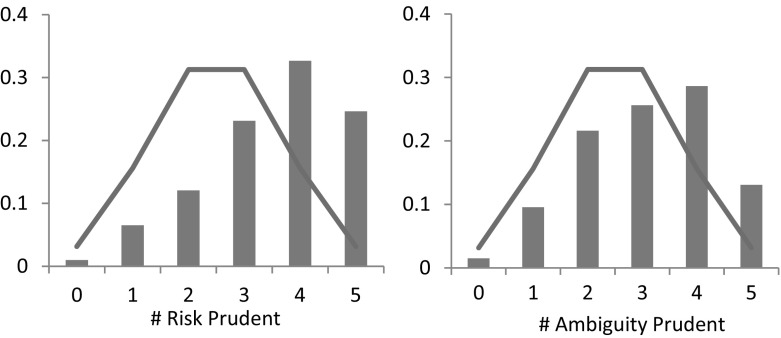
Distribution of risk- (*left*) and ambiguity- (*right*) prudent choices. *Note*: The solid line indicates the frequency with which a given number of choices would be expected to occur if each subjects chose randomly

Figure 8 presents the distribution of risk- and ambiguity prudent choices that we observed in the experiment based on 199 subjects, as well as the distribution that would be observed if subjects chose randomly (solid line).

First, we corroborate the common finding in the field that decision makers are risk prudent. On average, subjects chose the risk prudent option 3.54 out of 5 times, which is significantly different from the average that would be observed if subjects chose randomly (Wilcoxon signed-rank test, *p*-value < 0.01). In addition, the observed distribution of risk prudent choices is significantly different what would be observed if subjects chose randomly (*χ* ^2^ test, *p*-value < 0.01).

More interestingly, we confirm a strong preference for prudent options for ambiguity. On average, subjects chose the ambiguity prudent lottery 3.10 out of 5 times, which is significantly different from random choice (Wilcoxon signed-rank test, *p*-value = 0.01). In addition, the observed distribution of ambiguity prudent choices is significantly different what would be observed if subjects chose randomly (*χ* ^2^ test, *p*-value < 0.01). Thus, on average, subjects prefer to disaggregate a fixed probability loss and an informationally symmetric ambiguity, i.e., they can better cope with ambiguity if the wining probability is high, which implies that *φ*′′′ > 0 in the smooth model.

### Order 4: temperance

**Fig. 9:**
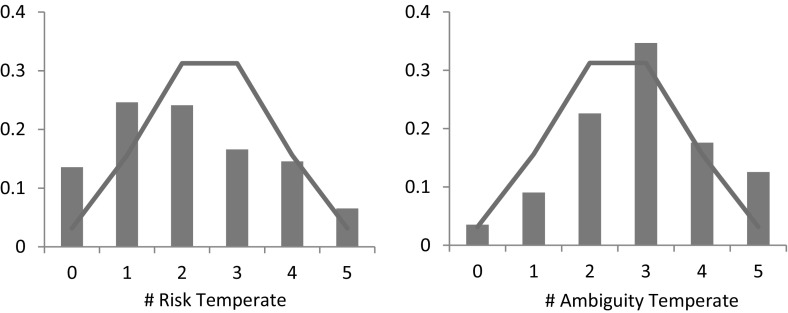
Distribution of risk- (*left*) and ambiguity- (*right*) temperate choices. *Note*: The solid line indicates the frequency with which a given number of choices would be expected to occur if each subjects chose randomly

Figure 9 presents the distribution of risk- and ambiguity temperate choices that subjects made. As the figure shows, we observe significant risk *in*temperance. Subjects chose the risk temperate lottery 2.14 times on average, which is significantly less than the average that would be expected if subjects chose randomly (Wilcoxon signed-rank test, *p*-value < 0.01). Moreover, the observed distribution of risk temperate choices is significantly different what would be observed if subjects chose randomly (*χ* ^2^ test, *p*-value < 0.01).

The observed preference for aggregating risks does not extend to ambiguity, as the right panel of Fig. 9 shows. On average, subjects chose the ambiguity temperate option 2.91 out of 5 times, which is significantly different from random choice (Wilcoxon signed-rank test, *p*-value < 0.01), and the observed distribution of ambiguity temperate choices is significantly different from the distribution that would be observed if subjects chose randomly (*χ* ^2^ test, *p*-value < 0.01). Our results thus indicate that subjects exhibit a preference to disaggregate independent informationally symmetric ambiguities, implying a negative fourth derivative of *φ* in the smooth model.

### Regression analysis

To investigate the determinants of higher order ambiguity attitudes, we run logistic regressions^4^ on choices, with a preference for Option A coded as 0 and for Option B coded as 1. Hence the regression results can be interpreted in terms of probability to choose the averse/prudent/temperate option. Our regression models include no constant, i.e., all coefficients equal to zero would imply random choice. Table 3 reports the results in terms of marginal effects.

**Table 3:** Analysis of the choices with logistic regressions, with and without control variables

	Model 1		Model 2		Model 3		Model 4		Model 5		Model 6	
Risk averse	0.36***	(0.02)	0.34***	(0.04)	0.40***	(0.04)	0.41***	(0.04)	0.35***	(0.04)	0.41***	(0.06)
Risk prud.	0.19***	(0.02)	0.18***	(0.02)	0.20***	(0.02)	0.19***	(0.03)	0.17***	(0.03)	0.17***	(0.05)
Risk temp.	−0.06***	(0.02)	−0.04*	(0.03)	−0.01	(0.03)	−0.01	(0.03)	−0.04	(0.03)	0.09*	(0.05)
Amb. avers.	0.08***	(0.02)	0.08***	(0.02)	0.07***	(0.02)	0.08***	(0.03)	0.10***	(0.03)	0.09**	(0.04)
Amb. prud.	0.11***	(0.02)	0.13***	(0.02)	0.12***	(0.02)	0.12***	(0.03)	0.13***	(0.03)	0.20***	(0.04)
Amb. temp.	0.07***	(0.02)	0.10***	(0.02)	0.09***	(0.02)	0.04	(0.03)	0.08***	(0.03)	0.11**	(0.04)
Impact of order (ambiguity first)												
Risk averse × order			0.03	(0.05)							0.03	(0.05)
Risk prud. × order			0.02	(0.04)							0.02	(0.04)
Risk temp. × order			−0.04	(0.04)							−0.05	(0.04)
Amb. avers. × order			0.01	(0.03)							0.01	(0.03)
Amb. prud. × order			−0.06*	(0.03)							−0.06**	(0.03)
Amb. temp. × order			−0.06**	(0.03)							−0.07**	(0.03)
Impact of the position												
Risk averse × pos.					−0.07	(0.06)					−0.07	(0.05)
Risk prud. × pos.					−0.02	(0.04)					−0.02	(0.04)
Risk temp. × pos.					−0.10***	(0.04)					−0.11***	(0.04)
Amb. avers. × pos.					0.02	(0.03)					0.02	(0.03)
Amb. prud. × pos.					−0.04	(0.03)					−0.04	(0.03)
Amb. temp. × pos.					−0.03	(0.03)					−0.03	(0.03)
Impact of gender (male)												
Risk averse × male							−0.08	(0.05)			−0.08	(0.06)
Risk prud. × male							−0.01	(0.04)			−0.01	(0.04)
Risk temp. × male							−0.09**	(0.04)			−0.09**	(0.04)
Amb. avers. × male							−0.01	(0.03)			0.01	(0.03)
Amb. prud. × male							−0.03	(0.03)			−0.02	(0.03)
Amb. temp. × male							0.05	(0.03)			0.05	(0.03)
Impact of study field (economics or finance)												
Risk averse × econ									0.02	(0.05)	0.04	(0.06)
Risk prud. × econ									0.03	(0.04)	0.03	(0.04)
Risk temp. × econ									−0.04	(0.04)	−0.03	(0.04)
Amb. avers. × econ									−0.03	(0.03)	−0.03	(0.03)
Amb. prud. × econ									−0.04	(0.03)	−0.04	(0.03)
Amb. temp. × econ									−0.02	(0.03)	−0.03	(0.03)
*χ* ^2^	411.98***		446.38***		446.02***		435.79***		424.77***		508.32***	
N	5970		5970		5970		5970		5940		5940	

Logistic regressions on choices, with a preference for Option A coded as 0 and for Option B coded as 1. The results can be interpreted in terms of probability to choose the averse/prudent/temperate option. There are no constant, i.e., all coefficients equal to zero imply random choice. The variable *order* is 1 if the subject started with ambiguity; *position* (*pos*.) is 1 if the averse/prudent/temperate option was on the left; *econ.* is 1 if the subject studied economics or finance. The table reports marginal effects, followed by standard errors (clustered at the individual level) between brackets

* *p* < 0.1

** *p* < 0.05

*** *p* < 0.01

In Model 1, we regress choices on binary variables capturing whether these choices concern aversion, prudence or temperance, under risk or under ambiguity. It confirms the result of the non-parametric analysis, in the sense that subjects prefer the averse/prudent/temperate option in all choices, except the risk temperate ones, where we observe risk intemperance. In Model 2, we interact the variables of Model 1 with a variable capturing whether the ambiguity part preceded the risk part or not. Starting with the ambiguity choices reduced ambiguity prudence and ambiguity temperance but did not reverse the choices. Model 3 shows that having the temperate option on the left made subjects choose it less often. Models 4 and 5 interact the variables of Model 1 with gender (being a male) and study field (studying economics or finance). It shows that the preference for risk intemperance is gender-specific: men are significantly intemperate. Thus, the often-observed gender difference in risk aversion, with men being inclined to take more risks than women (e.g., Croson and Gneezy 2009), appears to extend to temperance: compared to females, males exhibit a stronger preference towards aggregating independent zero mean risks. This result is consistent with Noussair et al. (2014), who observe that women are significantly more temperate than men in a representative sample of the Dutch population. Model 6 includes all variables. It confirms the effects previously described. The coefficient for risk temperance is positive (marginally significant) while it was negative for Model 1. This means that the reference category exhibited slight risk temperance but this concerns a very small sample.^5^

To further understand the determinants of choice behavior, we run a similar regression on the characteristics of the lotteries^6^ and the subjects (Model 7 in Table 4). In all the choices of the experiment, both options had the same expected value. However, the higher this shared expected value, the less likely the subjects were to choose the averse/prudent/temperate option. Unsurprisingly, subjects were more likely to choose these options, the higher the standard deviation of the alternative option. The third variable of Model 7 is the skewness of the averse/prudent/temperate option minus the skewness of the alternative option. The larger the skewness difference, the more subjects chose the averse/prudent/temperate option. This is consistent with preferring positively skewed options to negatively skewed ones, as observed by others (e.g., Ebert and Wiesen 2011). Finally, subjects disliked higher kurtosis. We found a small gender effect, and having the averse/prudent/temperate option displayed on the left made subjects choose it less. From Table 3, we know that these effects are mainly driven by temperance.

**Table 4:** Determinants of risk averse/prudent/temperate choices

	Model 7	
Expected value of both options	−0.01***	(0.00)
Standard deviation of alternative option	0.03***	(0.00)
Skewness difference	0.18***	(0.02)
Kurtosis difference	0.06***	(0.01)
Age	−0.00	(0.00)
Male	−0.05**	(0.03)
Studying economics or finance	0.01	(0.02)
Ambiguity first	−0.01	(0.02)
Averse/prudent/temperate option on the left	−0.07***	(0.02)
*χ*2	271.48***	
N	2970	

The table reports marginal effects of logistic regressions, followed by standard errors between brackets; skewness difference is the difference in skewness between the averse/prudent/temperate option and the seeking/imprudent/intemperate option; kurtosis difference is the difference in kurtosis between the seeking/imprudent/intemperate and the averse/prudent/temperate option

* *p* < 0.1

** *p* < 0.05

*** *p* < 0.01

### Mixed attitudes

To test the predictions regarding the link between the various orders of risk aversion and ambiguity aversion summarized in Table 1, we examine the behavior of risk- and ambiguity averters and risk- and ambiguity seekers separately. We classify subjects as follows. Those that choose the averse option 4 or 5 (0 or 1) out of 5 times are considered to be averse (seeking) for both risk and ambiguity. Under this classification, 10 subjects are classified as risk seeking and 157 are risk averse, while 24 subjects are classified as being ambiguity seeking and 70 subjects are ambiguity averse.

**Fig. 10:**
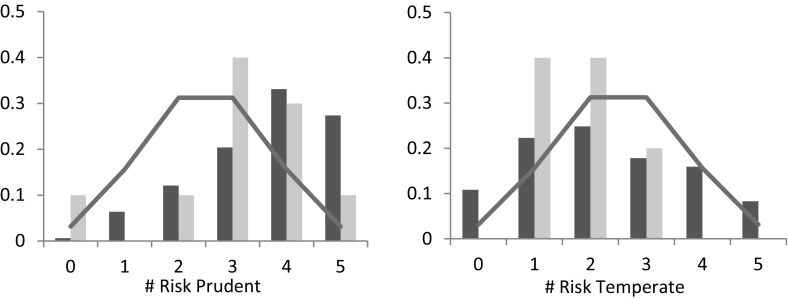
Distribution of choice behavior by risk type. *Note*: The solid line indicates the frequency with which a given number of choices would be expected to occur if each subjects chose randomly. Dark (light) bars represent risk averters (seekers)

Figure 10 shows the distribution of risk-prudent and risk-temperate choices for risk-seekers (light bars) and risk-averters (dark bars) separately. As the figure shows, the patterns revealed in the figure broadly replicate those found by Deck and Schlesinger (2014). That is, based on Wilcoxon signed-ranks tests, risk averters are significantly risk prudent (*p*-value < 0.01) while risk seekers are not significantly so (*p*-value = 0.12). However, risk averters are temperate neutral (*p*-value **=** 0.09), while risk seekers are risk intemperate (*p*-value = 0.03).

**Fig. 11:**
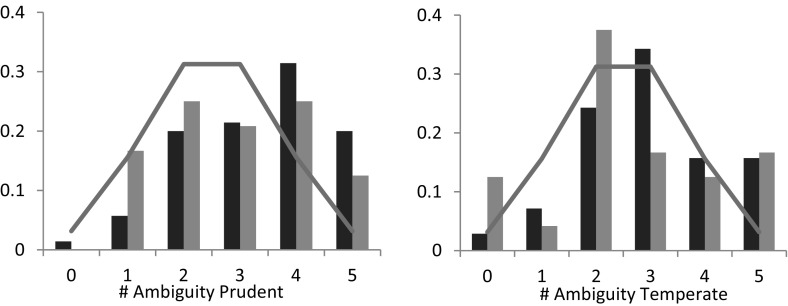
Distribution of choice behavior by ambiguity type. *Note*: The solid line indicates the frequency with which a given number of choices would be expected to occur if each subjects chose randomly. Dark (light) bars represent ambiguity averters (seekers)

A similar (mixed) pattern arises for ambiguity. Figure 11 shows the number of ambiguity prudent and ambiguity temperate choices for the 70 ambiguity averse subjects (dark bars) and 24 ambiguity seeking subjects (light bars) separately.

Based on Wilcoxon signed-rank tests, ambiguity averters are ambiguity prudent (*p*-value < 0.01), and ambiguity temperate (*p*-value < 0.01), while ambiguity seekers are ambiguity prudent neutral (*p*-value = 0.16) and ambiguity temperate neutral (*p*-value = 0.80).

Deck and Schlesinger (2014) and Ebert and Wiesen (2014) provide further evidence for mixed risk averse behavior by considering the correlation of individual behavior between tasks of different orders. In our sample, our measurement of the degree of risk aversion is indeed positively correlated with the degree of risk temperance (Spearman ρ = 0.22; *p*-value < 0.01), which corroborates the findings of Deck and Schlesinger (2014) and Ebert and Wiesen (2014). However, we also observe a significant positive correlation between the degree of risk prudence and the degree of risk temperance (Spearman ρ = 0.24; *p*-value < 0.01), which is not in line with the mixed risk aversion pattern. For ambiguity, we only observe a significant correlation between our measurement of ambiguity prudence and ambiguity temperance (Spearman ρ = 0.19; *p*-value < 0.01).

Even though ambiguity models are silent about the link between risk and ambiguity attitudes, one may wonder whether they are correlated: if subjects consistently combine good with bad for risk and ambiguity, such a correlation should be observed. Only the correlation between risk- and ambiguity aversion is significantly positive (Spearman ρ = 0.22; *p*-value = 0.002). It was not different from 0 for the higher orders.^7^ Previous empirical findings about the correlation between risk and ambiguity aversion are mixed. Most studies found positive correlations, a few negative correlations, and some no correlations at all (e.g., Sutter et al. 2013; Trautmann and van de Kuilen 2015).

To conclude, the evidence regarding the hypothesis that DMs consistently combine good with bad for ambiguity is mixed. Even though ambiguity averters are indeed ambiguity temperate as predicted by the pattern of mixed ambiguity aversion, we do not observe a significant correlation between the *degree* of ambiguity aversion and ambiguity temperance. A potential explanation for these results is the fact that the preference to combine good with bad is not consistent over probabilities; for unlikely events, the majority of subjects prefers to combine good with good, while for likely events we observe a consistent preference to combine good with bad. More research is needed to identify the link between a preference to combine good with bad and ambiguity attitudes.

## Discussion

In this study, we used an experiment to test for the prevalence of nonlinear preferences towards higher order ambiguity attitudes in a sample of 199 students. Our results show that the often-observed aversion towards ambiguity extends to prudence and temperance, which has important implications for the descriptive validity of assumptions underlying theoretical work on attitudes towards ambiguity.

Ambiguity, under which probabilities are unknown, opens up the possibility that results are affected by subjects’ beliefs. In the definitions presented in Sect. 3, we used the argument of informational symmetry, i.e., that subjects did not have any reason to expect one color to be more likely than the other if they were not explicitly told so. In ambiguity experiments, this concern is related to the risk of suspicion. A subject may suspect the experimenter to voluntarily put fewer cards of the winning color in the deck. To avoid suspicion, we made clear to subjects that they were free to change the winning color from red to black or two similarly exchange two ambiguous types of cards (kings and queens in Figs. 4b, 5b for instance). Only in 21% of all choices that option was ever chosen, suggesting that subjects were not especially suspicious, and that those that were suspicious did use the opportunity to exchange events.

Even though evidence in favor of risk prudence is strong and consistent across studies, evidence about risk temperance is mixed. Deck and Schlesinger (2010) and the present study observe intemperance, while other studies (e.g., Noussair et al. 2014; Deck and Schlesinger 2014) find evidence in favor of temperate behavior. A possible explanation for risk intemperance is probability weighting, for instance as exhibited in prospect theory (Tversky and Kahneman 1992) or rank-dependent utility (Quiggin 1981). In these models, small probabilities of high gains tend to be overweighed. For risk prudence tasks, this effect is likely to reinforce the preference for the prudent option, whose best outcome is always assigned a smaller probability than the best outcome of the imprudent option. For risk temperance, the opposite holds. By construction, the best outcome of lottery A_r4_ is assigned a smaller probability than the best outcome of lottery B_r4_. Hence, overweighting of small probabilities points towards risk prudence and risk intemperance.

An alternative explanation for the mixed findings regarding temperance is that the temperance preference is not that strong and, hence, more likely to be affected by framing effects. The studies that have observed a strong preference for temperate lotteries clearly frame the choice between temperate lotteries as a risk apportionment task, while we present the lotteries in their reduced form. Given the theoretical importance of risk temperance and the mixed results on its prevalence obtained in the laboratory, more research into framing effects on higher-order risk attitudes is warranted.

In our experiment, the evidence for ambiguity temperance is also rather weak. This could be explained by the complexity of the tasks, which involves many sources of ambiguity (e.g. color of the cards, Aces or Jacks, and Kings or Queens in Fig. 3). The task was more cognitively demanding and therefore more prone to error or random choice. From a theoretical perspective, ambiguity temperance has been little studied but it is predicted by models like multiplier preferences (Hansen and Sargent 2001). In real life, it may influence investment strategies in the presence of many sources of ambiguity. For instance, if there are two possible states of the economy and several companies whose performance is more ambiguous in one state than in the other, an ambiguity temperate DM will compose a portfolio that does not aggregate all ambiguities in one state.

Higher order preferences are defined by relatively complex preference conditions. Our experiment, being the first to explore this domain, was designed to remain as simple as possible. Hence, we restricted ourselves to binary choices in order to detect the presence of higher order preferences, and refrained from using valuation tasks. Our conclusions therefore only address the prevalence of higher order ambiguity preferences and not to their intensity (as for instance done for higher order risk preferences by Ebert and Wiesen 2014), which is left for future research.

## Conclusion

Since Ellsberg’s (1961) seminal paper, ambiguity aversion has been much studied, empirically and theoretically. Recent theoretical contributions had highlighted the role of higher order ambiguity preferences but it remained uncertain whether people actually exhibit such behavior. This paper is first to provide evidence of ambiguity prudence and ambiguity temperance. By doing so, it provided support for ambiguity models that predict ambiguity prudence. Some other ambiguity models are more general and do not specifically predict ambiguity prudence. Our results will help theorists fine-tune their models or help them choose specifications of those. For instance, in the smooth model, our results support choosing a smooth ambiguity function *φ* with a positive third derivative.

We also elicited higher risk attitudes and could analyze how each order of behavior correlates between risk and ambiguity and, within each domain, with the other orders. We found some, but limited, support for mixed behavior, i.e., the overall tendency to combine good with bad.

Finally, we have demonstrated how to measure ambiguity prudence and temperance. The tasks we introduced can be, for instance, added to survey on prevention behavior to better understand the role of ambiguity in prevention behavior.

## Electronic supplementary material

Below is the link to the electronic supplementary material.

Supplementary material 1 (PDF 393 kb)

## Footnotes

1 These results can be contrasted with those obtained for ambiguity aversion. Alary et al. (2013) provided conditions in which ambiguity aversion may actually decrease prevention efforts.

2 Throughout, we only consider lotteries leading to positive final wealth, to avoid bankruptcy considerations.

3 All Wilcoxon signed-rank tests reported in this section are based on the number of times subjects chose the averse/prudent/temperate option compared to 2.5. All Wilcoxon signed-rank test results are replicated with Fisher-Pitman permutation tests.

4 Clustering standard errors at the individual level. All results were the same with probit regressions (see Appendix C of electronic supplementary material).

5 It concerns female participants who did not study finance nor economics, started with the risk tasks and had the temperate option on the right of the screen.

6 The characteristics of the lotteries are reported in Appendix A of electronic supplementary material.

7 ρ = 0.01, *p* value = 0.89 for prudence; ρ = 0.06, *p*-value = 0.41 for temperance.
