# Supplementary material for: Measuring higher order ambiguity preferences
Source: Exp Econ. 2017 Aug 31;21(2):233–56. doi: 10.1007/s10683-017-9542-3 (PMC5913386; doi:10.1007/s10683-017-9542-3)
Supplement: Supplementary file 1 — Supplementary material 1 (PDF 393 kb) [file 10683_2017_9542_MOESM1_ESM.pdf]

Electronic supplementary material for
"Measuring Higher Order Ambiguity Preferences"

Aurélien Baillon
Erasmus University Rotterdam

Harris Schlesinger
University of Alabama

Gijs van de Kuilen
Tilburg University

Appendix A: Details about the risk tasks

Table A1: Characteristics of the Risk Tasks

Task	Order	EV	Standard deviation A	Standard deviation B	Skewness A	Skewness B	Kurtosis A	Kurtosis B
1	2	€15	15	0	0	0	1	1
2	2	€30	15	0	0	0	1	1
3	2	€22.5	22.5	0	0	0	1	1
4	2	€10	14.1	0	0.7	0	1.5	1
5	2	€20	12.6	0	-1.2	0	2.2	1
6	3	€11.25	6.5	6.5	-1.2	1.2	2.3	2.3
7	3	€26.25	6.5	6.5	-1.2	1.2	2.3	2.3
8	3	€22.5	13	13	-1.2	1.2	2.3	2.3
9	3	€7.5	5.6	5.6	-0.4	1.8	1.6	4.2
10	3	€17.5	9	9	-0.9	0.6	2.6	1.5
11	4	€15	7.5	7.5	0	0	4	1
12	4	€30	7.5	7.5	0	0	4	1
13	4	€22.5	11.3	11.3	0	0	4	1
14	4	€10	7.1	7.1	0.7	0.7	4.5	1.5
15	4	€20	7.1	7.1	-0.7	-0.7	4.5	1.5

Appendix B: Experimental Instructions

General Instructions

This experiment involves 30 choices between two options involving amounts of money and chance. At the end of the experiment, 1 of the choices will be carried out for real. One of you will now randomly draw a sealed envelope containing one of the choices (a number from 1 to 30). Thus, each choice has an equal chance to be selected. The envelope will be opened at the end of the experiment and the option that you have chosen in that particular choice will then be resolved and paid for real. On top of this payment, you will receive a show-up fee of €5, provided that you make all choices and complete a short questionnaire at the end of the experiment.

During the experiment:

- please no talking,
- turn off you cell phone,
- and raise your hand if anything is unclear, to be helped in private

We will wait for everybody to make each choice before proceeding to the next; you might have to wait a while before a new choice appears on your screen.

The experimenter will now hand out the instructions for the first 15 choices. The experimenter will read these instructions aloud. When everybody has completed the first 15 choices, instructions for the last 15 choices will be handed out.

Instructions for next 15 choices

The following 15 choices concern two options, called Option L (left) and Option R (right). All the options yield prizes depending on a card drawn from a particular deck of cards. This card will be drawn at random from the deck after the deck is thoroughly shuffled by a participant of the experiment. Thus, the experimenter cannot affect the card that is drawn in any way.

Before making each choice, you will receive information about the composition of the deck. For example, you might be informed that the prizes of two options depend on a card drawn from the example deck depicted below:

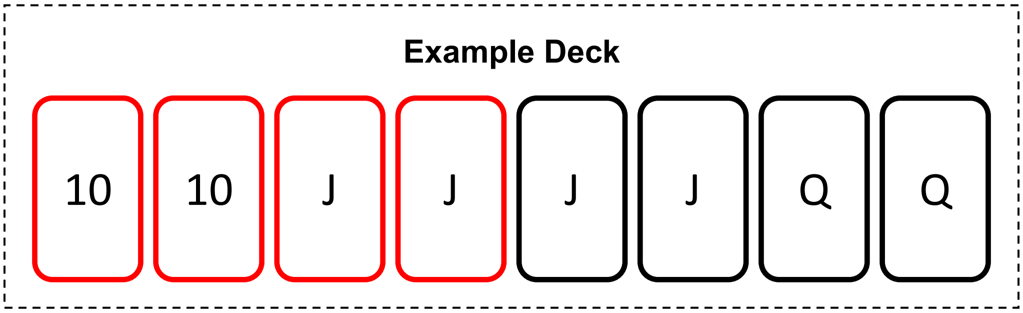

In this example, the deck consists of four red cars and four black cards. You can also see that there are two 10s, four Jacks (J), and two Queens (Q) in the deck.

As an example, consider the choice depicted below, based on a card drawn from the example deck:

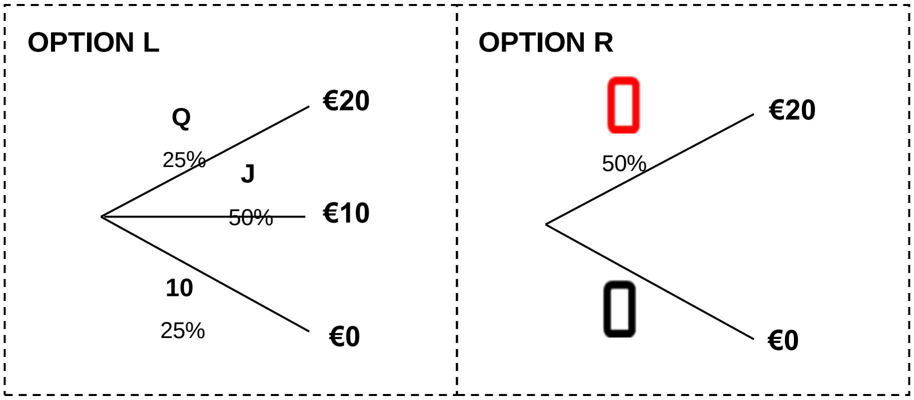

As you can see, Option L yields €20 if a Queen is drawn from the deck, €10 if a Jack is drawn from the deck, and nothing otherwise. Option R yields €20 if a red card is drawn from the deck, and nothing otherwise. As you can see, the resulting probabilities of yielding a prize are also depicted in both options. In this case, if you choose Option L, you have a 25% chance to win €20, a 50% chance to win €10, and a 25% chance to win €0. If you choose Option R, you have a 50% chance to win €20, and a 50% chance to win €0.

Note that in each choice, the *expected* payoff of both options is identical. In the example, the expected payoff is €10 for both options. However, the potential payoffs, and the chances to win these payoffs, differ between the options. This will be the case for the next 15 choices that you will encounter.

In the next 15 choices, you are asked to choose between similar options. Please raise your hand if you need further explanation from the experimenter. If not, the experimenter will soon start the program.

Instructions for next 15 choices

The following 15 choices concern two options, called Option L (left) and Option R (right). All the options yield prizes depending on a card drawn from a particular deck of cards. This card will be drawn at random from the deck after the deck is thoroughly shuffled by a participant of the experiment. Thus, the experimenter cannot affect the card that is drawn in any way.

Before making each choice, you will receive information about the composition of the deck. For example, you might be informed that for the resolution of two options in a particular choice a card will be drawn from the example deck depicted below:

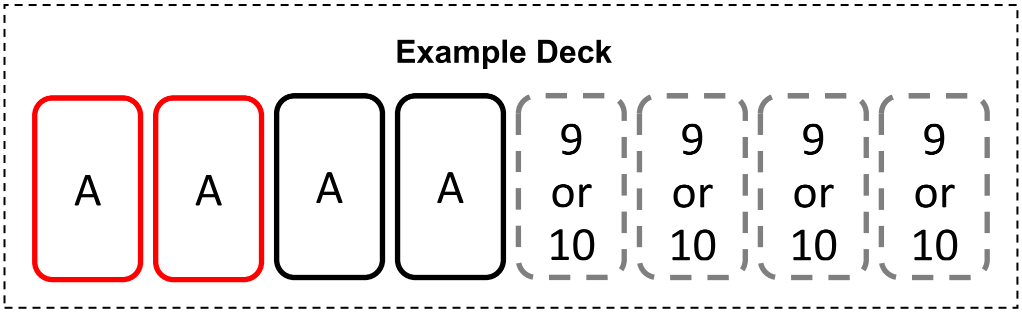

In this example, the deck consists of eight cards: two cars are red, two cards are black, and the color of four remaining cards in the deck is unknown, which is represented by the dashed line circumventing these four cards. The dashed line depicts the fact that these four cards will either **all be red or all be black**. Thus, the example deck either has six red cards (and two blacks cards) **or** six black cards (and two red cards).

You can also see that there are four Aces (A) in the deck. Each of the remaining cards is either a 9 or a 10. All combinations are possible: four 9s, three 9s and a 10, two 9s and two 10s, a 9 and three 10s, or four 10s.

As an example, consider the choice depicted below, based on a card drawn from the example deck:

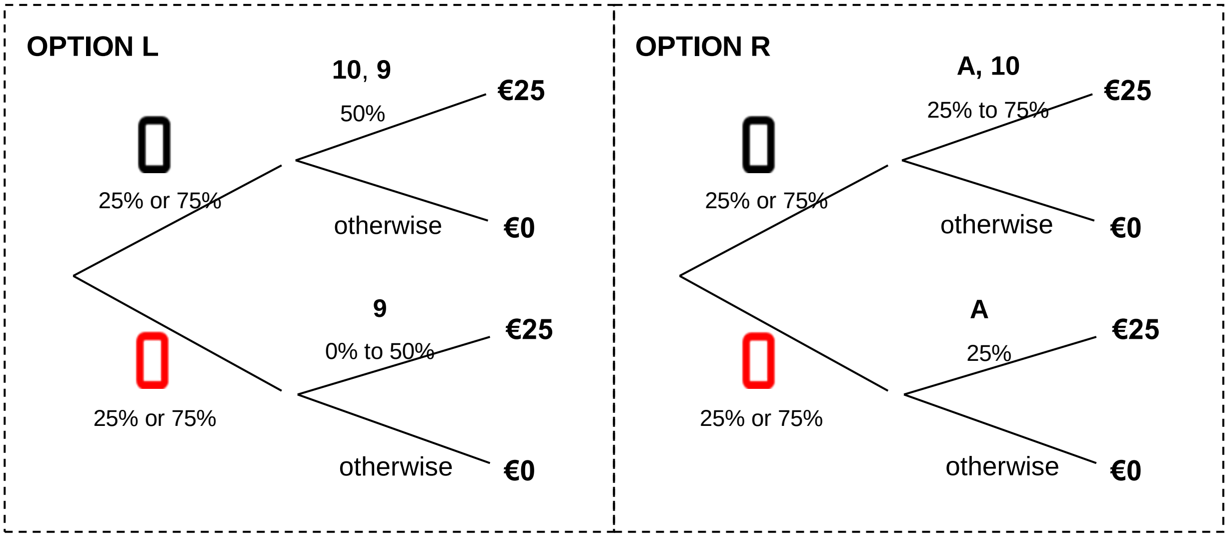

As you can see, Option L yields €25 if the card drawn from the deck is black and a 10 or a 9, or if the card is red and a 9. Otherwise, Option L yields nothing. Similarly, Option R yields €25 if the card drawn from the deck is black and an Ace or a 10, or if the card is red and an Ace. Otherwise, Option R yields nothing.

As you can see, the resulting probabilities of yielding a prize are also depicted in both options. In both options, the probability that a black card is drawn from the deck is either 25% (all the cards with an unknown color are red) or 75% (all the cards with an unknown color are black).

In Option L, if a black card is drawn from the deck, the probability of winning €25 is known to be 50%. If a red card is drawn in Option L, the probability of winning lies between 0% (all the unknown cards are 10s) and 50% (all the unknown cards are 9s).

In Option R, if a black card is drawn from the deck, the probability of winning lies between 25% (all the unknown cards are 9s) and 75% (all the unknown cards are 10s). If a red card is drawn in Option R, the probability of winning €25 is known to be 25%.

Moreover, you will have the possibility to exchange red and black in both options, and to exchange 9 and 10. If you do both, Option R would yield €25 if the card is red and an Ace or a 9, or if it black and an Ace.

In the next 15 choices, you are asked to choose between similar options. Please raise your hand if you need further explanation from the experimenter. If not, the experimenter will soon start the program.

Appendix C: Robustness check – Probit regressions

Table C1: Analysis of the choices with probit regressions, with and without control variables

	Model 1		Model 2		Model 3		Model 4		Model 5		Model 6	
Risk averse	0.35***	(0.02)	0.34***	(0.03)	0.39***	(0.04)	0.40***	(0.03)	0.34***	(0.04)	0.40***	(0.05)
Risk prud.	0.19***	(0.02)	0.19***	(0.02)	0.20***	(0.02)	0.20***	(0.03)	0.18***	(0.03)	0.18***	(0.05)
Risk temp.	-0.07***	(0.02)	-0.04*	(0.03)	-0.02	(0.03)	-0.01	(0.03)	-0.04	(0.03)	0.09*	(0.05)
Amb. avers.	0.08***	(0.02)	0.08***	(0.02)	0.07***	(0.02)	0.09***	(0.03)	0.10***	(0.03)	0.09**	(0.04)
Amb. prud.	0.11***	(0.02)	0.14***	(0.02)	0.13***	(0.02)	0.12***	(0.03)	0.13***	(0.03)	0.20***	(0.04)
Amb. temp.	0.07***	(0.02)	0.11***	(0.02)	0.09***	(0.02)	0.04	(0.03)	0.09***	(0.03)	0.12**	(0.05)
Impact of order (ambiguity first):												
Risk averse x order			0.03	(0.05)							0.03	(0.05)
Risk prud. x order			0.02	(0.04)							0.02	(0.04)
Risk temp. x order			-0.04	(0.04)							-0.05	(0.04)
Amb. avers. x order			0.01	(0.03)							0.01	(0.03)
Amb. prud. x order			-0.06*	(0.03)							-0.06**	(0.03)
Amb. temp. x order			-0.06**	(0.03)							-0.07**	(0.03)
Impact of the position:												
Risk averse x pos.					-0.06	(0.05)					-0.07	(0.05)
Risk prud. x pos.					-0.02	(0.04)					-0.02	(0.04)
Risk temp. x pos.					-0.10***	(0.04)					-0.12***	(0.04)
Amb. avers. x pos.					0.02	(0.03)					0.02	(0.03)
Amb. prud. x pos.					-0.04	(0.03)					-0.04	(0.03)
Amb. temp. x pos.					-0.03	(0.03)					-0.03	(0.03)
Impact of gender (male):												
Risk averse x male							-0.07	(0.05)			-0.07	(0.05)
Risk prud. x male							-0.01	(0.04)			-0.01	(0.04)
Risk temp. x male							-0.09**	(0.04)			-0.09**	(0.04)
Amb. avers. x male							-0.01	(0.03)			0.01	(0.04)
Amb. prud. x male							-0.03	(0.03)			-0.02	(0.03)
Amb. temp. x male							0.05	(0.03)			0.05	(0.04)
Impact of study field (economics or finance):												
Risk averse x econ									0.02	(0.05)	0.03	(0.05)
Risk prud. x econ									0.03	(0.04)	0.03	(0.04)
Risk temp. x econ									-0.04	(0.04)	-0.03	(0.04)
Amb. avers. x econ									-0.03	(0.03)	-0.03	(0.04)
Amb. prud. x econ									-0.04	(0.03)	-0.04	(0.03)
Amb. temp. x econ									-0.02	(0.03)	-0.04	(0.03)
Chi2	453.28***		493.80***		490.90***		484.99***		467.49***		565.97***	
N	5970		5970		5970		5970		5940		5940	

Notes: Probit regressions on choices, with a preference for Option A coded as 0 and for Option B coded as 1. The results can be interpreted in terms of probability to choose the averse / prudent / temperate option. There are no constant, i.e., all coefficients equal to zero imply random choice. The variable *order* is 1 if the subject started with ambiguity; *position (pos.)* is 1 if the averse / prudent / temperate option was on the left; *econ.* is 1 if the subject studied economics or finance. The table reports marginal effects, followed by standard errors (clustered at the individual level) between brackets. * $p < 0.1$, ** $p < 0.05$, *** $p < 0.01$

Table C2: Determinants of Risk averse/Prudent/Temperate Choices with probit regression

	Model 7	
Expected value of both options	-0.01^{***}	(0.00)
Standard deviation of alternative option	0.03^{***}	(0.00)
Skewness difference	0.17^{***}	(0.02)
Kurtosis difference	0.05^{***}	(0.01)
Age	-0.00	(0.00)
Male	-0.05^{**}	(0.03)
Studying economics or finance	0.01	(0.02)
Ambiguity first	-0.01	(0.02)
Averse/prudent/temperate option on the left	-0.07^{***}	(0.02)
Chi2	271.48^{***}	
N	2970	

Note: The table reports marginal effects of probit regressions, followed by standard errors between brackets; * p < 0.1, ** p < 0.05, *** p < 0.01; skewness difference is the difference in skewness between the averse/prudent/temperate option and the seeking/imprudent/intemperate option; kurtosis difference is the difference in kurtosis between the seeking/imprudent/intemperate and the averse/prudent/temperate option.
